# Protocol for a feasibility study to inform the development of a multicentre randomised controlled trial of asthma-tailored pulmonary rehabilitation versus usual care for individuals with severe asthma

**DOI:** 10.1136/bmjopen-2015-010574

**Published:** 2016-03-23

**Authors:** Sally Majd, Lindsay D Apps, Nicky Hudson, Stacey Hewitt, Elizabeth Eglinton, Anna Murphy, Peter Bradding, Sally Singh, Ruth Green, Rachael Evans

**Affiliations:** 1 Centre for Exercise Rehabilitation Science, Leicester, UK; 2 Leicester Respiratory Biomedical Research Unit, Glenfield Hospital, Leicester, UK; 3 Infection, Immunity and Inflammation, University of Leicester, Leicester, UK; 4 School of Applied Social Sciences, DeMontfort University, Leicester, UK; 5 Patient Representative, Leicester, UK; 6 Department of Respiratory Medicine, Thoracic Surgery and Allergy, Glenfield Hospital, Leicester, UK; 7 School of Pharmacy, DeMontfort University, Leicester, UK

**Keywords:** Severe asthma, Pulmonary rehabilitation, Exercise, QUALITATIVE RESEARCH

## Abstract

**Introduction:**

Pulmonary rehabilitation with core components of exercise training and multiprofessional education is an integral part of the management of patients with chronic lung disease. International guidelines for individuals with asthma recommend exercise as exercise improves symptoms, indices of cardiopulmonary efficiency, health status and psychosocial outcome. However, there is little published evidence evaluating safety and acceptability of exercise training for individuals with severe asthma and there are concerns regarding exercise-induced asthma. We propose a feasibility study for a multicentre randomised controlled trial (RCT) of asthma-tailored pulmonary rehabilitation (asthma-tailored PR) versus usual care in individuals with severe asthma.

**Methods and analysis:**

The study will be conducted in three stages. Adults with severe asthma will be included if they have persistent symptoms despite being at step 4 or 5 of the British Thoracic Society guidelines. Stage 1: semistructured interviews will be used in a sample of 20–30 individuals with severe asthma to understand the experience and attitudes of this population towards exercise. Stage 2: eight focus groups of at least six healthcare professionals involved in the care of patients with severe asthma will be conducted to understand their attitudes towards exercise for this population. Stage 3: a small-scale RCT of the proposed multicentre RCT of asthma-tailored PR versus usual care for individuals with severe asthma will be conducted. The primary outcome measures will be recruitment, retention and adverse event rates. Semistructured interviews with participants of stage 3 will be used to identify further barriers or facilitators to participation in PR and the trial. Thematic analysis will be used for the interpretation of all interviews.

**Ethics and dissemination:**

The study results will inform the design of a larger multicentre RCT. The National Research Ethics Service Committee East Midland approved the study protocol.

**Trial registration number:**

ISRCTN96143888.

**Strengths and limitations of this study**

- This study focuses on the feasibility of a planned multicentre randomised controlled trial of asthma-tailored pulmonary rehabilitation versus usual care for severe asthma.
- The intervention will be tailored using information from qualitative studies regarding the views around exercise and exercise programmes of both patients with severe asthma and healthcare professionals involved in their care.
- The success of the study will depend on developing appropriate recruitment strategies.

## Introduction

The most recent global estimate of asthma suggests that up to 334 million people suffer from asthma with a high burden of disability^1^ that is estimated to be greater than for both diabetes and breast cancer.^2^ Much of this disability is in the 10–20% of patients with severe disease despite currently available therapies^3^ ^4^ who consume 50–60% of the healthcare costs attributed to asthma.^5^ ^6^ This reflects a considerable unmet need for this population, and represents a major economic burden. Novel approaches to treatment are therefore needed urgently.

Cardiorespiratory fitness is a strong predictor of mortality in both health and disease.^7^ Improving cardiorespiratory fitness through regular exercise reduces the prevalence of many diseases which are common in patients with severe asthma^8^ and importantly reduces the risk of premature death.^9^ Unfortunately, asthma has a strong independent inverse association with cardiorespiratory fitness,^10^ and exercise-induced dyspnoea and activity limitation are very common in patients with asthma^11^ ^12^ leading to a fear of exercise.^13^ A progressive loss of cardiorespiratory fitness (deconditioning) leads to a reduction in the threshold for exercise-induced symptoms and a downward spiral of inactivity. Exercise can induce bronchoconstriction in some individuals with asthma, which is usually a reflection of uncontrolled asthma, but maybe an overperceived concern for patients and healthcare professionals alike. In one study, hyperventilation was the commonest cause of exercise limitation in patients with difficult asthma, whereas exercise-induced bronchoconstriction was present in <10%.^14^

Structured physical training is a key component of pulmonary rehabilitation (PR) programmes (alongside education and self-management). PR has shown consistent benefits for patients with chronic obstructive pulmonary disease (COPD) and other chronic lung diseases.^15^ The principles are to target the extrapulmonary manifestations including peripheral muscle dysfunction^16^ and mood disturbance.^15^ There are limited data available on skeletal muscle function in asthma, but the pathological mechanisms in severe disease maybe similar to those in patients with COPD, principally inactivity, deconditioning, inflammatory and steroid use. A Cochrane review^17^ concluded that physical training improves cardiorespiratory fitness in people with asthma similar to that seen in non-asthmatic individuals, with no evidence that exercise had adverse effects on lung function or wheeze however, the majority of participants were children with mild-to-moderate asthma. Two studies in adults with moderate asthma suggested that compared with controls, supervised exercise training improves symptoms, health-related quality of life (HRQoL), anxiety and depression and aerobic capacity.^18^ ^19^ Unfortunately similar benefits were not replicated following a 12-week self-directed exercise programme suggesting that supervision was a key factor for a positive outcome.^20^ A further randomised controlled trial (RCT) reported no adverse events with exercise training in moderate-to-severe asthma.^21^ However, there were limited data regarding the applicability to adults with severe or refractory asthma as the majority of study participants had moderate asthma based on the Global Initiative for Asthma criteria.^22^

Data from animal models of asthma^21^ ^23^ suggest that exercise not only improves cardiorespiratory fitness, but also directly decreases eosinophilic airway inflammation. Subsequently, this has been shown in human adults with moderate-to-severe asthma where a significant reduction in eosinophilic airway inflammation was seen in those receiving aerobic training, breathing exercises and education compared with a control group receiving breathing exercises and education only.^21^ A systematic review on the effect of physical training (moderate-intensity aerobic exercise) on airway inflammation in adults with asthma^24^ suggested physical training may decrease airway inflammation, but owing to reporting issues, lack of information and heterogeneity, there was no definite conclusion.

Obesity is prevalent in people with asthma, which may contribute to the pathogenesis, and weight loss is associated with improved asthma control in this group.^25^ A differential effect has been suggested between weight loss achieved through either diet or exercise on neutrophilic or eosinophilic airway inflammation, respectively, in obese individuals with asthma.^26^

In an earlier study, we surveyed 60 patients with severe asthma^27^ and only 25% reported physical activity within recommended levels, while 65% had stopped exercising due to asthma symptoms, 87% wanted to be fitter and 72% were interested in participating in an exercise programme. Despite this, patients with severe asthma are often excluded from community-based exercise referral schemes due to perceived higher risks by providers and patients, and perhaps some healthcare professionals. For example, patients with more severe disease are more likely to believe that exercise is not good for asthma.^13^ To date, self-directed exercise has been unsuccessful in this group.^20^ The UK Department of Health suggests that patients with severe chronic disease require highly adapted, supervised exercise programmes.^16^ All these factors may cause potential barriers to exercise in patients with severe asthma.

Standard pulmonary rehabilitation might be beneficial for this patient population. Previously 111 patients with a diagnosis of severe asthma completed our local PR programme designed for patients with COPD. Statistically significant improvements were seen in dyspnoea, anxiety and depression scores, HRQoL and exercise capacity^28^ (the latter was assessed by the incremental shuttle walk test (ISWT) also successfully used for the aerobic training exercise prescription). Although the results of PR were positive, there was probably some bias in the referral pattern of our clinicians; patients were not typical of our severe asthma population as they were older with a COPD phenotype. Importantly, discussions with patients with severe asthma^27^ suggest most would prefer to participate in an exercise programme tailored specifically towards asthma. Patients would also prefer to exercise near home, but for safety reasons we initially propose to evaluate a hospital-based programme. The James Lind Alliance^29^ has identified physical therapy, psychological interventions and self-management as key areas for research identified for asthma by patients. We therefore propose a feasibility study of a RCT of asthma-tailored PR compared with usual care (UC) for individuals with severe asthma. We will explore both patient and healthcare professional attitudes towards exercise in severe asthma. The data from these studies will be used to inform the adaptation of current pulmonary rehabilitation for this population, and assess the feasibility of a multicentre trial of asthma-tailored PR for severe asthma.

## Methods and analysis

### Study design

This feasibility project has been designed using the Medical Research Council guidelines on developing complex interventions.^30^ It will have three stages and adopts a mixed-methods approach with separate recruitment for each stage.

### Participants

Participants for stages 1 and 3 will be recruited from a population of patients under physicians specialising in the care of patients with difficult-to-treat asthma at Glenfield hospital, Leicester, UK, and will have had a thorough diagnostic assessment and appropriate treatment strategy instituted prior to recruitment. They will be identified as eligible if they have:

- Symptomatic asthma despite being on step 4–5 treatment according to SIGN/BTS guidelines^31^ (high-dose inhaled corticosteroids (>1000 µg beclomethasone equivalent) plus a second controller and/or systemic corticosteroids);
- Been treated under the care of a difficult-to-treat asthma specialist for at least 6 months.

Participants will be excluded if they:

- Have a diagnosis of smoking-related COPD;
- Have both fixed airflow obstruction (FEV_1_/FVC <70%) and a smoking history of ≥10 pack years;
- Are unable to exercise—for example, due to significant musculoskeletal or neurological abnormality;
- Have a history of significant cardiac disease—for example, myocardial infarction (MI) within the last 3 months, unstable angina or heart failure, and/or significant valvular disease;
- Had a severe exacerbation of asthma in the preceding month prior to entry to the programme;
- Had a hospital admission due to an exacerbation of asthma within the last 3 months;
- Had an ITU admission involving intubation within the last year.

Informed written consent from potential participants will be obtained by the research team.

### Stage 1

#### Understanding the barriers and facilitators to exercise in individuals with severe asthma

The aims of this qualitative stage are to understand the experience of individuals with severe asthma and their attitudes towards physical activity, exercise and structured exercise programmes. For the current study, the data will inform:

- Approaches to recruitment;
- How to overcome the barriers to engaging in exercise and develop strategies to promote positive exercise behaviours;
- The proposed topics and self-management strategies for the education component of the PR programme;
- Acceptability and therefore feasibility of a structured programme.

##### Study design

A descriptive study will be undertaken using semistructured interviews. Basic demographic information will be collected and the following patient characteristics to be represented have been identified (based on previous discussion with patient representatives, the literature^13^ ^32^ ^33^ and the research team): age, gender, social class, obesity, employment status, dependents, living arrangements and transport availability. Participants will be recruited until both theoretical saturation and the diversity of the sample required has been met; a sample of approximately 20–30 patients is anticipated.

Audio-recorded interviews will be conducted privately face-to-face between the participant and an interviewer. Interviews will be transcribed verbatim by a professional transcriber with identifiable information removed. Interview questions have been devised based on relevant literature, experience of the team and consultation with patient representatives. They will be piloted before use to ensure validity. Interviews will be analysed using thematic analysis^34^ supported by NVivo software (V.10, QSR International). This approach follows six distinct stages: familiarisation with data, generating initial codes, searching for themes, reviewing themes, defining and naming themes and producing the report. Initial coding will be carried out and a sample of interviews will be coded by a second member of the team to ensure consistency and to enhance interpretive authenticity. Throughout the data analysis, the team will meet to discuss and review emerging themes and search for accounts that provide contesting views of the same phenomena or identify different phenomena. Our patient representative will be invited to comment on our (anonymised) initial findings to ensure interpretations made by researchers stay close to the direct experience of patients.^35^

### Stage 2

#### Understanding healthcare professionals attitudes towards exercise as a therapy for severe asthma

The aim of this stage is to explore healthcare professionals’ perspectives on exercise and exercise programmes. This data set will be used to inform:

- The design of the education for the asthma-tailored PR programme;
- Understand referral behaviours and identify any potential barriers from healthcare professionals to promoting exercise;
- Recruitment strategies (including information given to GPs) for the current study and the multicentre trial;
- Dissemination strategies.

##### Study design

Eight focus groups of 6–10 healthcare professionals^36^ involved in the care of patients with severe asthma will be conducted. A purposive sampling strategy will be employed to ensure a range of professions and seniority is represented including physiotherapists, asthma nurses, pharmacists, respiratory nurses, general respiratory physicians, occupational therapists, general practitioners (GPs) and managers. Personnel will be recruited from a tertiary referral centre (n=1), district general hospitals (n=3) and local GP practices (n=4) to fully explore views across different settings. The focus groups will be held in the respective settings to maximise participation. Group discussions will be facilitated by a researcher experienced in qualitative research and will encourage the exchange of consensus and disagreements on participation in exercise and exercise programme for this population.

Focus groups will be audio-recorded and transcribed as per stage 1. Methodology for data analysis will be as described for stage 1, but will pay close attention to the additional complexity and interaction inherent in focus group data.^37^ A sample of transcripts will be analysed by all members of the research team to ensure agreement over emerging themes.

### Stage 3

#### A small-scale version of an RCT trial of asthma-tailored PR versus UC in individuals with severe asthma

##### Study design

**Figure 1:**
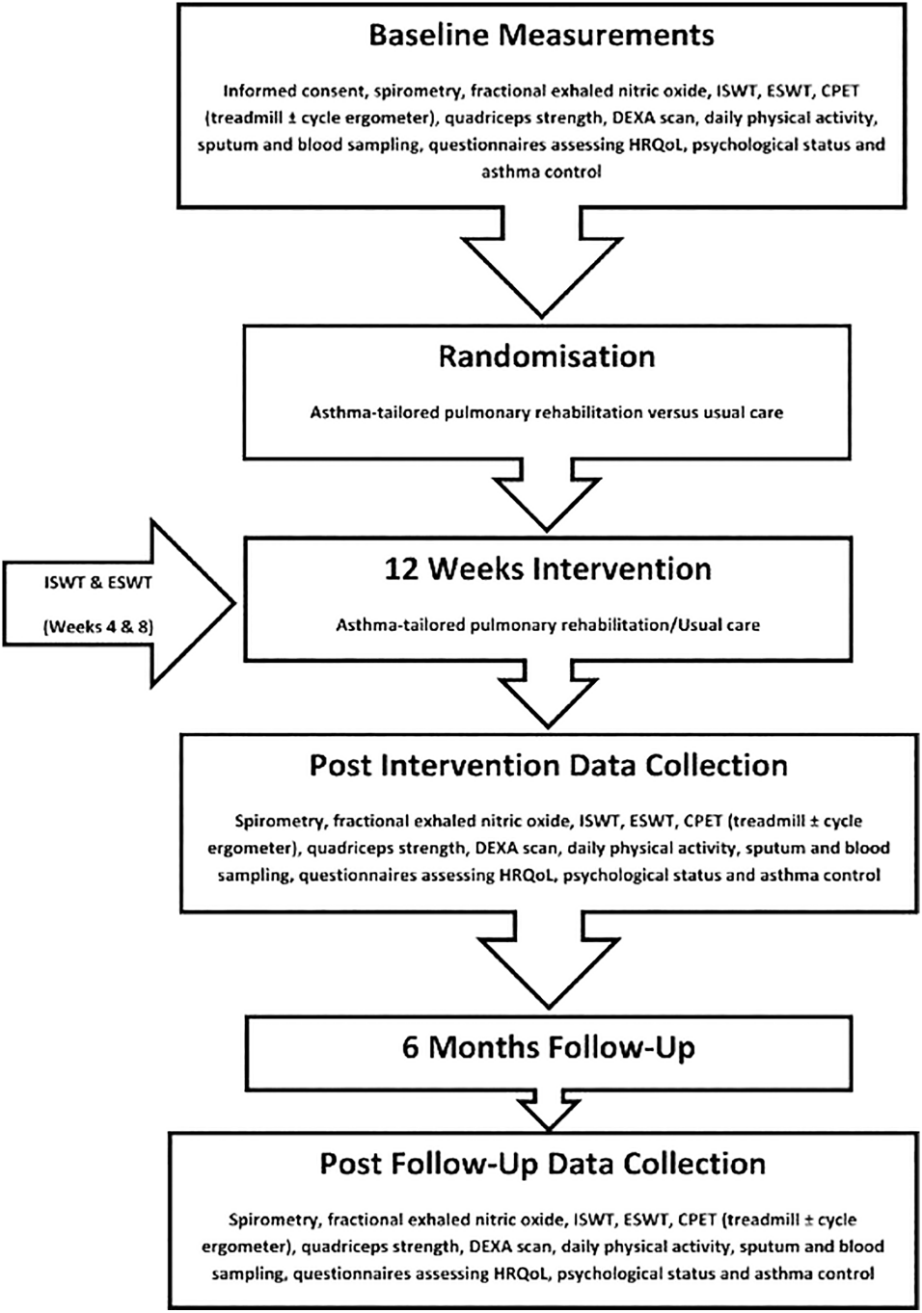
Study timetable.

A small-scale RCT will be conducted to assess feasibility of a large multicentre trial. A maximum of 60 patients will be recruited. Participants will be randomised to either the asthma-tailored PR group or UC group using internet-based ‘sealed envelope’ randomisation codes as advised by our local clinical trials unit. Randomisation to treatment group allocation will be a 2:1 ratio (asthma-tailored PR:UC) as retention rate is the primary outcome rather than effectiveness. Randomisation allocation is sent by automated email to unblinded research team members (data will be collected and analysed prior and immediately post intervention and at 6-month follow-up (figure 1)).

##### Intervention (asthma-tailored PR)

Asthma-tailored PR will be based on a traditional PR programme but will be exclusively for severe asthma. It will be undertaken in a hospital setting in case of adverse events. The programme will extend over 12 weeks with two supervised, 2 h sessions per week (1 h of exercise and 1 h of multidisciplinary education) and a well-defined home exercise programme. A rolling programme will be used and the duration of the programme may have to be extended for return after exacerbations.

For the exercise component, combined endurance and strength training will be based on our local successful PR programme and evidence-based international guidelines for PR and health.^38^ ^39^ An exercise prescription includes intensity, frequency, mode and duration, but there is little specific guidance for asthma^38^ ^40^ other than high-intensity aerobic training^41^ (60–80% of peak oxygen consumption (VO_2_ peak) or peak heart rate). We propose predominantly high-intensity walking-based endurance training, as our local preliminary data were successful.^28^ We will aim for an initial walking duration between 6 and 10 min to be increased to 20–30 min over 1–4 weeks aiming for an end Borg Score for breathlessness of 4–6.^42^ The speed of the walk will be recalibrated at 4 and 8 weeks, where needed. It is anticipated that fast walking will be high-intensity exercise for this population, but cycling or jogging will be prescribed if needed. A home walking diary will be kept and participants advised to walk daily. Strength training will comprise upper and lower limb exercises: two sets of 6–12 repetitions at 80% one repetition maximum for knee extensors and flexors on the gym equipment and elbow flexion and extension using free weights. The programme above is a preliminary design and any part of this will be adapted for the feasibility study according to the results of stages 1 and 2.

Half of the educational component will comprise a series of sessions facilitated by the multidisciplinary team. These will be based on the current PR education programme but will be developed to be more specific to patients with asthma. There will be some modification after stages 1 and 2 regarding the topics and content of the sessions. The other half of the education sessions will be based on motivational consultation delivered by a health psychologist and related to discussion within the group about modifying behaviour, how to become more active, exercise diary review, setting and resetting goals.

##### Usual care

All participants will be seen by physicians specialising in the care of patients with difficult-to-treat asthma. They will receive standard asthma management including disease education provided by experienced asthma nurse specialists; specific advice regarding participation in regular exercise will be given. They will be offered standard PR after participation in the trial.

##### Outcome measurements

The primary outcome measures are:

- Recruitment rate (incorporating willingness of patients to be randomised and practicality of a hospital-based programme);
- Retention rate (incorporating acceptability of the therapy);
- Incidence of adverse events.

We will record the time to recruit and complete the trial.

The secondary outcome measures are:

- Exercise capacity assessed by both laboratory treadmill testing (and optional cycle ergometer),^43^ (including exercise-induced bronchoconstriction^44^ and dynamic hyperinflation) and field testing by both ISWT^45^ and the endurance shuttle walk test (ESWT).^46^ The repeatability of the ISWT in this population will also be assessed.
- Sputum eosinophil count,^47^ exhaled nitric oxide and inflammatory markers in the blood.
- Asthma control using the Asthma Control Questionnaire (ACQ).^48^
- HRQoL using the Asthma Quality of Life Questionnaire (AQLQ)^49–51^ and Chronic Respiratory Questionnaire.^52^
- Psychological morbidity using the Hospital Anxiety and Depression Scale.^48^
- Quadriceps strength assessed by an adapted chair with a strain gauge^53^ and body composition assessed by dual-energy X-ray absorptiometry (DEXA).^53^
- Domestic physical activity measured by tri-axial accelerometers (Sensewear pro 3).^52^

The outcome measures to assess the intervention will be collected at baseline, 12 weeks and 9 months by a blinded investigator (see figure 1 for study schedule). Baseline demographics will be recorded including body mass index (BMI) and spirometry. To promote participant retention and follow-up, all the study visit dates and times will be arranged at the participant's convenience during the first visit. They will receive a reminder telephone call the day before their visit. In the event of participant's withdrawal, any data collected prior to that point will be reported.

##### Safety

- Any adverse events directly or indirectly related to the exercise measurements and training sessions will be recorded. Any severe adverse event will be reported to our research and development office immediately. An external respiratory physician has agreed to independently assess any adverse events and stop the trial, if necessary
- A full cardiopulmonary exercise test with expiratory gas analysis will be performed on a treadmill.^43^ Exercise-induced bronchoconstriction will be assessed by a flow-volume loop, before and after the treadmill test. This data will be used as an outcome measure not just for safety
- Asthma control and disease activity will be assessed by the ACQ, measuring airway inflammation by induced sputum count before and after the intervention and by comparing the preceding 9 months unscheduled healthcare visits for asthma with 9 months after and including the intervention including GP, accident and emergency attendance and hospital admissions

##### Cost-effectiveness

The EuroQol^54^ questionnaire will be used to assess quality-adjusted life-years (QALYs) at baseline, 12 weeks and 9 months. Costs will be calculated using NHS tariffs. All treatment including medication, adverse events and any over-the-counter medication will be recorded.

##### Exacerbation of asthma

Patients will be asked to keep a diary of their exacerbations, treatment and unscheduled healthcare visits. The patients will be asked to stop coming to the asthma-tailored PR sessions for any exacerbation requiring steroid treatment. If they are not recovered enough to start participation within 2 weeks, they will be classified as dropout. Similarly, they will be excluded from the UC limb if they are not back to normal within 2 weeks to avoid bias.

##### Sample size calculation

The primary outcomes for the feasibility study are recruitment, retention and incidence of adverse events, to establish the practicality of a definitive multicentre RCT. We plan to recruit 40 patients to the intervention arm and 20 patients to UC. We estimate conservatively that we will recruit 30% of those invited to estimate the recruitment rate with a precision of at least ±7%. We suggest a conservative dropout rate of 25% (our local PR dropout rate is approximately 15%); recruiting 40 patients to the asthma-tailored PR programme, the precision of the estimated retention rate would be at least ±14%.^55^ From experience to date, we are expecting very few serious adverse events relating to the exercise programme. Based on a rate of 2.5%, the rate would be estimated to be less than 13%.^56^ All precisions are based on two-sided 95% CIs.

##### Statistical analysis

Data collected will be entered to a secured local database by the researchers. A descriptive analysis will be performed using mean (SD) and median (IQR) for normally and not normally distributed variables, respectively. The recruitment and retention rates will be described as percentages.

## Ethics and dissemination

### Monitoring and oversight

We have organised a steering group consisting of an external investigator, patient representatives, statistician and the coinvestigators of the project who will meet three times a year to review the safety and progress of the project.

The principle and coinvestigators will have access to the local data set with password-protected access.

#### Dissemination

The results of this feasibility study will inform the planning of a definitive RCT, including whether it can be done, by assessing rates of recruitment, retention, serious adverse events and data collection. The qualitative and physiological analysis will be used to further refine the asthma-tailored PR programme and the study protocol for the proposed multicentre trial. The participants will be encouraged to have a role in the design of the definitive trial. The findings of the study and the subsequent proposal for the multicentre RCT will be presented to potential collaborators including British Thoracic Society Severe Asthma registry along with local GPs, respiratory nurse specialists and medical colleagues through presentations at local educational events. We will also disseminate the findings of the feasibility study and the proposed RCT to the charity Asthma UK and to our local asthma and pulmonary rehabilitation PPI advisory groups before seeking funding for the substantive multicentre study from the HTA or Asthma UK. The results of the feasibility study (stages 1–3) will be presented at local, national and international respiratory meetings as well as in manuscripts submitted to peer-reviewed journals.
